# Isocaloric Dietary Changes and Non-Alcoholic Fatty Liver Disease in High Cardiometabolic Risk Individuals

**DOI:** 10.3390/nu9101065

**Published:** 2017-09-26

**Authors:** Giuseppe Della Pepa, Claudia Vetrani, Gianluca Lombardi, Lutgarda Bozzetto, Giovanni Annuzzi, Angela Albarosa Rivellese

**Affiliations:** Department of Clinical Medicine and Surgery, Federico II University, 80131 Naples, Italy; gdp0206@libero.it (G.D.P.); c.vetrani@libero.it (C.V.); lombardi.gian@gmail.com (G.L.); lutgarda.bozzetto@unina.it (L.B.); annuzzi@unina.it (G.A.)

**Keywords:** NAFLD, NASH, isocaloric dietary changes, carbohydrates, monounsaturated fatty acids, polyunsaturated fatty acids, polyphenols, vitamins

## Abstract

Non-alcoholic fatty liver disease (NAFLD) incorporates an extensive spectrum of histologic liver abnormalities, varying from simple triglyceride accumulation in hepatocytes non-alcoholic fatty liver (NAFL) to non-alcoholic steatohepatitis (NASH), and it is the most frequent chronic liver disease in the industrialized world. Beyond liver related complications such as cirrhosis and hepatocellular carcinoma, NAFLD is also an emerging risk factor for type 2 diabetes and cardiovascular disease. Currently, lifestyle intervention including strategies to reduce body weight and to increase regular physical activity represents the mainstay of NAFLD management. Total caloric intake plays a very important role in both the development and the treatment of NAFLD; however, apart from the caloric restriction alone, modifying the quality of the diet and modulating either the macro- or micronutrient composition can also markedly affect the clinical evolution of NAFLD, offering a more realistic and feasible treatment alternative. The aim of the present review is to summarize currently available evidence from randomized controlled trials on the effects of different nutrients including carbohydrates, lipids, protein and other dietary components, in isocaloric conditions, on NAFLD in people at high cardiometabolic risk. We also describe the plausible mechanisms by which different dietary components could modulate liver fat content.

## 1. Introduction

For its critical position between the systemic circulation and the blood flow of the gastrointestinal tract mediated by the portal vein, the liver plays an essential role in the intermediary metabolism, transforming dietary nutrients into the major chemical elements crucial for life and human health. Conversely, many nutrients and the overall dietary composition can influence liver function. In fact, an excessive intake of refined carbohydrate and saturated fats, the increased consumption of fructose and other simple sugars, and the progressive diffusion of high-calorie Western diets, the deleterious eating habits typical of the last forty years, have been associated with a dramatic increase in overweight/obesity and insulin resistance and, more recently, also with non-alcoholic fatty liver disease (NAFLD) [1]. Noteworthy, the excess of adiposity, in particular abdominal adiposity, and insulin resistance are the major contributors to the development of several cardiometabolic abnormalities strictly related to the increased risk of cardiovascular disease (CVD) and type 2 diabetes mellitus (T2DM). Interestingly, NAFLD itself can be considered as an independent cardiometabolic risk factor beyond the classical cardiometabolic risk factors [2].

NAFLD is characterized by an excessive accumulation of lipids in the liver, primarily in the form of triglycerides, in the absence of a considerable alcohol ingestion (ethanol intake ≤ 30 g/day for men and ≤20 g/day for women), and ruling out other causes of liver injury [3]. The term NAFLD incorporates an extensive spectrum of histologic liver abnormalities, varying from simple triglyceride accumulation in hepatocytes non-alcoholic fatty liver (NAFL) or steatosis to non-alcoholic steatohepatitis (NASH), characterized by the additional presence of inflammation and tissue injury [4].

### 1.1. NAFLD and Cardiometabolic Risk

NAFLD is the most common chronic liver disease in the industrialized world with a 15–30% prevalence reported in the general population [5]. In particular, the prevalence of NAFLD is very high in individuals at high cardiometabolic risk. Cardiometabolic risk refers to a condition strongly associated with an increased risk of developing CVD and T2DM as a consequence of the presence of interrelated alterations in metabolic and vascular functions, as well as of dyslipidemia, hypertension, abdominal obesity, insulin resistance and hyperglycemia. All these abnormalities identify the metabolic syndrome; consequently, the close association between NAFLD and metabolic syndrome is unsurprising [6,7]. In line with these observations, the prevalence of NAFLD is approximately 50% in hypertensive subjects, 70% in people with T2DM, and up to 90% in severely obese patients [8,9,10]. Dramatically, NAFLD is also the most prevalent form of chronic liver disease in childhood and very recent data indicate that nearly 70–80% of obese children may have NAFLD [11]. Given the increasing prevalence of obesity and metabolic syndrome, NAFLD will become one of the most important public health challenges in the next decades for its related complications. In particular, it should be considered that simple NAFLD can progress to NASH in about 20–25% of cases, and nearly 20% of patients with NASH can develop fibrosis and cirrhosis [12]; in patients with cirrhosis, the cumulative incidence of hepatocellular carcinoma ranges from 2.4% to 12.8% over 3–7 years [13]. Beyond the liver-related complications, it is important to underline that NAFLD is also an emerging risk factor for T2DM and CVD [14,15], and that it has recently been associated with an increased risk of chronic kidney disease [16].

### 1.2. Pathogenesis of NAFLD

The mechanisms involved in NAFLD development and progression are not completely clear. The hypothesis of the “two-hit” model for the first time proposed by Day et al. in 1998 in the pathogenesis of NAFLD has been accepted for about one decade [17]. According to this model, the “first hit” is characterized by the accumulation of lipids primarily in the form of triglycerides derived from esterification of free fatty acids and glycerol in the hepatocytes. [18]. In particular, Donelly et al. clearly observed that 59% of the triglycerides present in the liver of patients with NAFLD derived from free fatty acids released from adipose tissue, 26% from de novo lipogenesis, and 15% from dietary lipids [19]. The low rate of β-oxidation of free fatty acids and the reduction in triglyceride export by very low density lipoprotein particles in a liver with increased fat content is also important [20]. Insulin resistance plays a pivotal role in the “first-hit” and in liver triglyceride accumulation. Increasing the free fatty acids release from adipose tissue, reducing the glucose uptake from the skeletal muscle and favoring the hepatic influx of these metabolites; furthermore, insulin resistance increases de novo lipogenesis and reduces the synthesis and secretion of very low density lipoprotein [21].

The increase in liver triglyceride content is strongly associated with hepatocyte susceptibility to the damage promoted by the “second hit”. The “second-hit” can be promoted by lipid peroxidation, oxidative stress, inflammatory cytokines, and mitochondrial dysfunction. All together, these factors induce steatohepatitis and can lead to fibrosis, which can evolve into cirrhosis [22].

In the last few years, based on a large body of knowledge, the hypothesis of the “two-hit” model has been translated into the “multiple-hit” model. In fact, it appears reasonable that the simple “two hit” mechanism is too reductive and inadequate to explain the complex mechanisms involved in NAFLD development and progression; furthermore, only a minority of patients with NAFLD progress to NASH or cirrhosis [1], and, on the other hand, steatohepatitis can precede simple steatosis [23].

The “multiple-hit” model provides a comprehensive model that takes into account the multiple factors and interactions involved in NAFLD [24]. Based on this model, dietary habits, insulin resistance, visceral adiposity, inflammatory state, oxidative stress, alteration in microbiome, and genetic predisposition, are all recognized risk factors for NAFLD development and progression.

**Figure 1:**
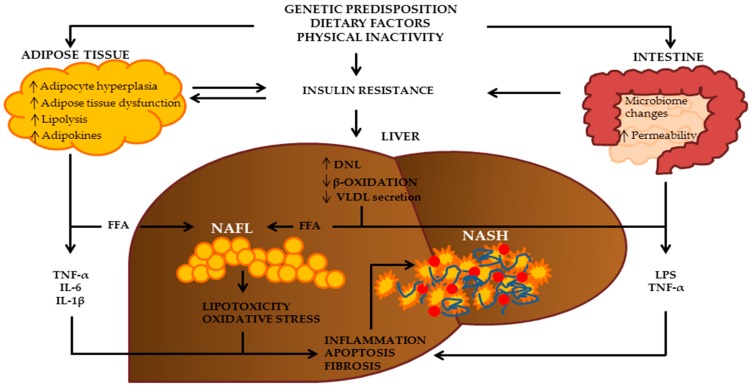
Pathogenesis of NAFLD. Based on the “multiple hit” model, dietary habits, insulin resistance, visceral adiposity, inflammatory state, oxidative stress, alteration in microbiome, and genetic predisposition, are all recognized risk factors for NAFLD. DNL: de novo lipogenesis; FFA: free fatty acids; IL-6: interleukin-6; IL-1β: interleukin-1β; LPS: lipopolysaccharide; NAFLD: non-alcoholic fatty liver disease; NASH: non-alcoholic steatohepatitis; TNF-α: tumor necrosis factor-α.

In particular, the type of diet, other environmental factors and genetic predisposition play an important role in the development of insulin resistance, visceral obesity, and gut microbiome changes. Insulin resistance promotes steatosis with the mechanisms above discussed; adipose tissue is involved beyond the free fatty acids efflux in the production and secretion of the inflammatory cytokines and adipokines involved in NAFLD progression [25]. Changes in the gut microbiome related to dietary habits can influence energy homeostasis and systemic inflammation [24]; all these factors can aggravate oxidative stress and endoplasmic reticulum stress in hepatocytes, leading to hepatic inflammation [26]. Furthermore, genetic predisposition of single nucleotide polymorphisms in genes such as Patatin-Like Phospholipase 3 (PNPLA3) or in Transmembrane 6 Superfamily Member 2 (TM6SF2) can aggravate liver injury [27] (Figure 1).

With respect to the strong relation between genetic predisposition and dietary habits, NAFLD represents an optimal example of disease by which nutrigenomics has allowed us to understand how nutrients can influence its development and progression by altering the expression of genes involved in inflammation, glucose and lipid metabolism [28]. Nutrigenomics focuses on identifying and understanding molecular interactions between nutrients/dietary bioactive compounds with the genome [29]. With regard to NAFLD, the PNPLA3 I148M polymorphism is a clear example of these possible interactions: individuals with the PNPLA3 I148M polymorphism are more prone to develop steatosis when the intake of carbohydrates, in particular simple sugars, is elevated [30]. Briefly, PNPLA3 exerts a lipolytic activity on triglycerides and its up-regulation is mediated by carbohydrates [31]; in individuals with the PNPLA3 I148M polymorphism, the high intake of carbohydrates induces the accumulation of the pathological protein less able to hydrolyze the triglycerides on the surface of lipid droplets and a consequent decreased secretion of triglyceride-rich lipoproteins from the liver [32]. Based on these observations, individualized nutritional strategy considering also the genetic features of individuals may be more effective in clinical management of NAFLD.

### 1.3. Diagnosis of NAFLD

Liver biopsy is still the gold standard for the diagnosis of NAFLD, and this invasive procedure despite some limitation related to sampling variability and procedural potential risk discerns simple NAFL from NASH [33]. However, in large population assessment or for disease monitoring, some non-invasive markers have been proposed. In terms of biochemical markers, it should be considered that serum levels of alanine aminotransferase (ALT) and aspartate aminotransferase (AST) are inaccurate markers of NAFLD [33].

For NAFL evaluation the best validated scores are represented by: the fatty liver index [34], the NAFLD liver fat score [35] and the Steato test [36], based on some biochemical markers and clinical parameters. In terms of instrumental evaluation, the first-step is represented by ultrasonography (US) [37,38], although it is limited by the possible interference of liver fibrosis on bright liver echo pattern and the very low sensitivity and specificity in individuals with BMI > 40 kg/m^2^. CT presents similar accuracy for NAFLD as US; however, it is limited by radiation exposure [33]. The proton magnetic resonance spectroscopy (^1^H-MRS) can reveal a liver fat content as low as 1%, but it is limited by its high cost [33]. In terms of biochemical markers, cytokeratin-18 (CK-18) fragments, derived from hepatocytes apoptosis or death, are only modestly accurate; therefore, for the diagnosis of NASH, liver biopsy is still the only diagnostic procedure [39]. Several scores based on biochemical parameters have been proposed to evaluate liver fibrosis as NAFLD Fibrosis Score, Fibrosis 4 Calculator, Enhanced Liver Fibrosis, and the Fibro Test [40]. Transient elastography is the instrumental imaging performed for the evaluation of liver fibrosis, but it has a high rate of false positive results [41].

### 1.4. Management of NAFLD: Hypocaloric Diet and Physical Activity

At present, lifestyle intervention including strategies to reduce body weight and increase regular physical activity represents the mainstay of NAFLD management. Recently, the Clinical Practice Guidelines for the management of NAFLD proposed by a joint effort of the European Association for the Study of the Liver, the European Association for the Study of Obesity, and the European Association for the Study of Diabetes recommended a 7–10% body weight loss in overweight/obese patients with NAFLD as a target to achieve [33,42]. A similar target is proposed by the American Association for the Study of Liver Diseases [2].

Body weight loss in NAFLD can be achieved by hypocaloric diet alone or in combination with increased physical activity.

Although total calorie intake plays a very important role in both the development and the treatment of NAFLD, only modulating the quality of the diet, i.e., changing either the macro or the micronutrient composition, can also markedly affect the clinical evolution of NAFLD offering a more realistic and feasible treatment alternative. To this regard, the Mediterranean diet characterized by high consumption of olive oil as source of added fat, legumes, whole grains, fruits, vegetables, and fish; a low consumption of dairy products and meat; and a moderate alcohol assumption [43] could represent an adequate therapeutic approach in NAFLD prevention and treatment and this dietary pattern has been recently recommended as good for the management of NAFLD [33].

The beneficial effect of Mediterranean diet on many metabolic chronic disease is largely supported by several epidemiological studies [44]. To date, some observational studies and few clinical trials have investigated the effects of Mediterranean diet on NAFLD; recently, Zelber-Sagi et al. have comprehensively reviewed the evidence on this aspect [45]. Nine studies are reported by the authors three cross sectional studies, one case-control study and five intervention trials and the adherence to the Mediterranean diet was significantly related to an improvement of NAFLD specific outcomes evaluated, i.e., reduction in liver enzymes in one study, reduction in liver fat content evaluated by ^1^H-MRS in three studies and by US in two studies, reduction in the fatty liver index in one study, and a lower grade of steatosis in the two studies a cross-sectional and a case-control, respectively, in which liver biopsy was performed [45].

The aim of the present review is to summarize the current evidence available on the effects of different nutrients, lipids, carbohydrates, protein and other dietary components on NAFLD in people at high cardiometabolic risk, in particular individuals with abdominal obesity and other metabolic abnormalities as well as dyslipidemia, hypertension, and hyperglycemia.

We have comprehensively evaluated the evidence from randomized controlled trials performed in humans to study the effects of different dietary components on NAFLD considering biochemical markers, liver scores, liver imaging US, ^1^H-MRS or computed tomography (CT) or liver biopsy as possible outcomes. It is important to underline that we have reported and discussed only trials evaluating the effects of nutrients in isocaloric conditions, i.e., the dietary regimen was designed to be neutral in energy balance compared with control. For *n*-3 polyunsaturated fatty acids (PUFA), vitamins and other bioactive dietary components, randomized controlled trials with dietary supplementation were considered. We also describe the plausible mechanisms by which different dietary components could modulate liver fat content.

## 2. Diet Composition and Non-Alcoholic Fatty Liver Disease in Isocaloric Conditions

### 2.1. Dietary Fatty Acids

In NAFLD as well as in other conditions (insulin resistance, blood lipids, etc.), the quality of different fatty acids seems to be more important than the total amount. Therefore, we will consider the different types of dietary fat separately.

#### 2.1.1. Saturated Fatty Acids

Saturated fatty acids (SFA) contain no double bonds in the straight-chain hydrocarbon with varying length ranging from short chain length (volatile liquids) to chain lengths of 30 or more carbon atoms (waxy solids). The main food sources are animal fat products such as cream, cheese, butter, other whole milk dairy products and fatty meats and eggs, but also some vegetable fat, i.e., coconut and palm kernel oils. The most consumed SFA are myristic (C14:0), palmitic (C16:0) and stearic (C18:0) acids.

Observational studies focusing on dietary habits of patients with NASH have suggested the possible negative influence of SFA, since their diets were richer in SFA than in other fatty acids compared to subjects with simple liver steatosis [46] or to the general population [47].

Along this line, controlled intervention studies demonstrated that increasing dietary SFA in isocaloric substitution of carbohydrates [48] or PUFA [49] increased hepatic and visceral fat accumulation in healthy subjects.

The detrimental effect of SFA on liver fat may be mediated by the increase in insulin resistance and oxidative stress, both associated with NAFLD. To date, studies in vitro and in animal models have shown that SFA could induce lipogenesis by promoting the transcription of peroxisome proliferator-activated receptor-gamma (PPAR-γ) coactivator-1β and the sterol regulatory element-binding transcription factor 1c (SREBP-1c). In addition, they promote oxidative stress, and apoptosis of hepatocytes [50,51], possibly leading to the progression of NAFLD to NASH [52].

#### 2.1.2. Monounsaturated Fatty Acids and *n*-6 Polyunsaturated Fatty Acids

Monounsaturated fatty acids (MUFA) contain one double bond in their aliphatic hydrocarbon chain. MUFA are mainly found in plant-based foods such as olive oil, canola oil, nuts, soy and avocado, and to a lesser extent in red meat and whole milk products. The greatest source of dietary MUFA (~90% of all MUFA) is oleic acid (C18:1 *n*9), followed by palmitoleic acid (C16:1 *n*9).

PUFA contain more than one double bond in their chemical structure. There are two main PUFA groups with relevant biological functions and they are classified by the position of their first double bond counting from the methyl carbon: *n*-6 PUFA with their first unsaturated bond at carbon6 and *n*-3 PUFA at carbon 3. The most biologically relevant *n*-6 PUFA are linoleic acid (LA, 18:2) and arachidonic acid (AA, 20:4); their main dietary sources are flaxseed and some nuts.

Albeit scant, the evidence available to date shows quite clearly the effectiveness of MUFA on liver fat (Table 1). After an eight-week intervention with a high-MUFA diet (28% of total energy) vs. a high-carbohydrate/high-fiber/low-glycemic index diet (MUFA 16% of total energy), a 29% reduction of liver fat content, measured by ^1^H-MRS, was observed in a group of T2DM subjects without body weight changes in comparison to a baseline diet moderately rich in SFA (13% of total energy) [53].

An even greater reduction (−39%) was observed in only six weeks by Ryan and colleagues [54] in a group of T2DM subjects with NAFLD. The participants were assigned to an isocaloric Mediterranean diet (MUFA intake 23% of total energy) where the main MUFA sources were extra-virgin olive oil, olives and nuts, whereas the control diet was a low-fat/high-carbohydrate diet (MUFA intake 8% of total fat). In a long-term intervention trial (24 weeks) [55], olive oil (MUFA 70%) and canola oil (MUFA 61%) consumption was compared to control oil (soybean or safflower oil such as the most common oil used in the habitual diet, MUFA 15–24%) in a group of Indian men. The two active groups showed a remarkable reduction of fatty liver grading evaluated by US, with 66.7% and 76.7% of the participants in the olive and canola oil groups, respectively, reverting to normal liver grading after the intervention.

**Figure 2:**
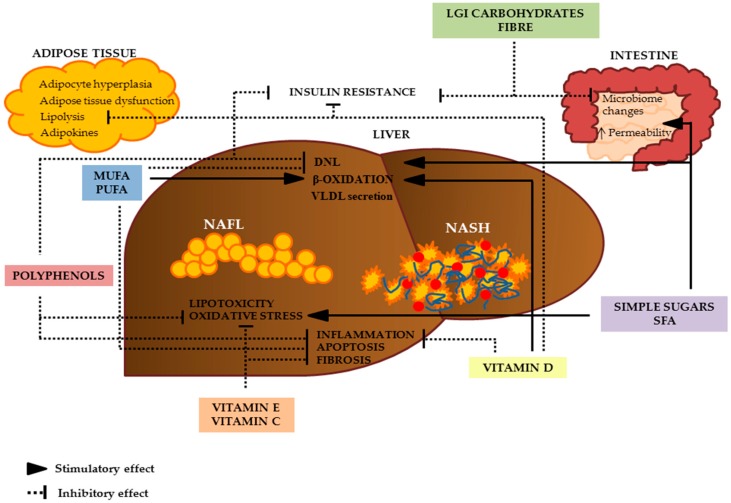
Possible sites of action of dietary nutrients in the nutritional treatment and prevention of NAFLD. Nutrients and dietary composition can modulate many key aspects in the pathophysiology of NAFLD: simple sugars promote DNL, produce inflammation and activate cellular stress pathways. Contrarily, LGI meals can improve insulin resistance and can positively modulate the microbiome. SFA could induce lipogenesis, oxidative stress, and apoptosis of hepatocytes; conversely, MUFA and PUFA can improve FFA β-oxidation and can reduce DNL, improve insulin sensitivity and reduce inflammation. Polyphenols could inhibit DNL and increase FFA β-oxidation. Furthermore, polyphenols can improve insulin sensitivity, reduce the transcription of inflammatory cytokines, and can mitigate the oxidative stress involved in NAFLD progression. Vitamin C and vitamin E could avoid the progression of NAFLD and improve NASH acting as powerful antioxidants; furthermore, vitamin E could reduce plasma levels of cytokines involved in inflammation and liver fibrosis. Vitamin D can reduce the transcription of inflammatory cytokines and improve FFA β-oxidation. Furthermore, it has been observed that vitamin D increases adiponectin secretion, decreases lipolysis in adipose tissue, and improves insulin resistance. DNL: de novo lipogenesis; LGI: low glycemic index; MUFA: monounsaturated fatty acids; NAFLD: non-alcoholic fatty liver disease; NASH: non-alcoholic steatohepatitis; PUFA: polyunsaturated fatty acids; SFA: saturated fatty acids.

Although the evidence is rather convincing, the exact mechanism through which MUFA could affect hepatic triglycerides content is not completely clear. In both in vitro and in vivo studies, MUFA have been shown to activate peroxisome proliferator-activated receptor-alfa (PPARα) and PPARγ [56], increasing lipid oxidation [57,58,59] and inhibiting lipogenesis [58,60], thus leading to a reduction in hepatic steatosis (Figure 2). On the other hand, MUFA can promote fatty acid deposition in adipose tissue rather than in the liver, enhancing the clearance of circulating triglyceride rich lipoproteins by lipoprotein lipase [61]. In addition, clinical studies suggest that the beneficial effects of MUFA on NAFLD may be driven also by an improvement in blood lipid profile (reduction of triglycerides, VLDL, LDL and oxidized-LDL, and an increase in HDL), insulin resistance and obesity-related inflammation [62,63].

As for the effect of *n*-6 PUFA on NAFLD, only one study met our inclusion criteria (Table 1). In a 10-week isocaloric randomized and controlled trial [64], participants were assigned either a PUFA diet or a saturated fat diet. Fat quality difference was obtained by the selection of specific foods: sunflower oil and seeds for the PUFA diet (linoleic acid 15%), and butter for the saturated fat diet group. After the intervention, liver steatosis assessed by ^1^H-MRS was significantly reduced with the PUFA diet compared to the SFA diet (−26% vs. +8%, respectively). In this study as well as in other studies with MUFA, no effects on liver enzymes were observed and no liver biopsies were performed.

Therefore, we can conclude that MUFA and *n*-6 PUFA seem to have beneficial effects on liver fat content in individuals at high cardiometabolic risk.

As for the possible mechanisms, PUFA are key regulators of the transcription of genes associated with lipid metabolism and mitochondrial β-oxidation (i.e., PPAR-α and SREBP-1). Thus, increasing PUFA intake may lead to a reduction of lipogenesis in favor of an increased hepatic fatty oxidation [65] (Figure 2).

#### 2.1.3. *n*-3 Polyunsaturated Fatty Acids

As reported above, *n*-3 PUFA is one of the two main PUFA groups with relevant biological functions. The most biologically relevant *n*-3 PUFA are α-linolenic acid (ALA, 18:3), eicosapentaenoic acid (EPA, 19:5) and docosahexaenoic acid (DHA, 22:6). The main dietary sources of *n*-3 PUFA are fish oil, flaxseed and some nuts; however, DHA and EPA are synthetized from ALA. Several studies on *n*-3 PUFA supplementation and NAFLD are available in individuals at high cardiometabolic risk. Overall, the available evidence still produces conflicting results (Table 2).

In a 24-week intervention trial, a complete fatty liver regression was observed after a 2 g/day of *n*-3 PUFA supplementation in the context of an American Heart Association (AHA) diet in 33.4% of the patients. The daily energy intake of the ADA diet was composed of 50% carbohydrates, 20% protein and 30% fat, and an eating pattern including a variety of fruits and vegetables, whole grain products, fat-free and low-fat dairy products, fish, peas, poultry and lean meats was recommended [66]. Interestingly, there was a significant improvement in the echogenicity score in the whole active group (AHA diet +2 g/day *n*-3 PUFA) as compared with the control group (AHA diet alone), the former displaying also a decrease in ALT concentration. These findings were further confirmed by the results of the WELCOME study [67]. The supplementation with 4 g/day of PUFA (1 g containing 460 mg of EPA and 380 mg of DHA) over 18 months significantly affected liver fat content in a dose-dependent manner (1% DHA enrichment in erythrocytes was associated with a 3% reduction in liver fat percentage) as compared with placebo.

Increasing the amount of *n*-3 PUFA to 6 g/day in the context of an AHA diet, Zhu et al. [68] detected a full reversion of liver steatosis in 19.7% of their patients, and an overall improvement of fatty liver grading in 53% of the study population after a 24-week intervention with no adverse events in patients who completed the treatment. In addition, ALT decreased more significantly in the treatment group than in the placebo group.

In contrast with the above studies, an eight-week supplementation with 9 g/day of fish oil (51.4% EPA and 23.9% DHA) vs. placebo did not affect hepatic triglyceride content measured by ^1^H-MRS [69]; similarly, Argo et al. [70] did not detect any improvement of fatty liver or inflammation, ballooning, or fibrosis scores in a group of subjects receiving 3 g/day of fish oil (35% of EPA and 25% of DHA) for 12 months as compared with the placebo group. On the same line, a 12-month supplementation with EPA (1.8 mg/day or 2.7 g/day) had no significant effects on the key features of NASH (i.e., fibrosis, lobular inflammation, and hepatocyte ballooning) [71].

Cussons and colleagues [72] compared the effects of the daily consumption of *n*-3 PUFA or MUFA in a group of women with polycystic ovary syndrome, a condition associated with NAFLD. According to an eight-week crossover randomized and controlled trial, they consumed 4 g/day of *n*-3 PUFA (56% DHA and 27% EPA) and a placebo (4 g/day, 67% oleic acid). Both arms reduced liver fat measured by hepatic ^1^H-MRS (*n*-3 PUFA: 18.2% vs. MUFA: 14.8%).

As reported above, the effectiveness of *n*-3 PUFA supplementation on liver fat content is still controversial. This lack of concordance may be due, at least in part, to the largely different doses used in the trials (ranging from 0.25 to 6 g/day), the length of the exposure (from 2 to 18 months), and finally the imaging methods (US vs. ^1^H-MRS). Nevertheless, the only two studies looking at NASH features on liver biopsies showed no effect of *n*-3 PUFA.

To date, evidence of the mechanisms linking *n*-3 PUFA supplementation and NAFLD derives mainly from in vitro and animal studies. First of all, as reported for *n*-6 PUFA, increasing PUFA intake may increase fatty oxidation in the liver through the modulation of PPAR-α and SREBP-1 [65]. On the other hand, EPA and DHA are important modulators of the inflammatory pathway and, consequently, may inhibit pro-inflammatory eicosanoid production by inflammatory cells related to hepatic injury in NAFLD (Figure 2).

### 2.2. Carbohydrates

In the last few years, clear evidence has emerged that carbohydrates quality is more important than quantity in determining metabolic effects, and this seems particularly relevant for the possible effect on liver fat. Therefore, we evaluate the effects of the quality of carbohydrates on liver fat especially in terms of glycemic index (GI) and simple sugars.

#### 2.2.1. Low Glycemic-Index Carbohydrate and Fiber Rich Diets

The association among high carbohydrate intake, high GI carbohydrate consumption, insulin resistance and liver fat accumulation has been found in animal models and observational studies [73,74]. In particular, in a cross-sectional study, the prevalence of high-grade liver steatosis increased significantly across quartiles of high GI versus low GI diets [75]. In fact, available carbohydrates produce an increase in serum levels of glucose that can be used for the synthesis of new triglycerides through de novo lipogenesis in the liver [76]. The consumption of foods with high GI promotes insulin resistance, a condition strongly related to NAFLD [77], and the negative effect of a high GI diet on liver fat content can be observed in few days [78]. Conversely, low GI meals could have beneficial effect on NAFLD. In fact, low GI foods, especially foods rich in fiber, can decrease glucose absorption, reducing hepatic influx of glucose and de novo lipogenesis [79]; in addition, the fiber content of low GI foods can positively act on the gut microbiome, a possible mediator by which nutrients may influence liver fat content [80] (Figure 2).

Although GI seems to be an important factor in NAFLD prevention and treatment, few clinical trials have investigated the effect of low GI or low glycemic load (GL) at isocaloric conditions on NAFLD in patients at high cardiometabolic risk (Table 3).

It is important to underline that, in all of the above studies, the diets utilized were different not only for GI but also for other dietary components which could influence per se the parameters evaluated. Three studies evaluated the effects of low GI diets on liver fat compared to diets with higher GI, and two of them found a significant reduction in liver fat evaluated by ^1^H-MRS in one and by US in the other [81,82]; no change was observed in the third study performed in obese children [83]. In none of these three studies was there any change with respect to liver enzymes; on the other hand, a reduction in ALT was reported after a low GI diet and a Mediterranean diet compared to a control diet in one intervention trial performed in patients with T2DM in which only liver enzymes were analyzed [84].

The fiber content of foods is one of the most important factors related to GI. Dietary fiber is defined as a non-digestible food ingredient; based on solubility it can by classified into soluble pectins, fructans, oligosaccharides and gums and insoluble hemicellulose, cellulose and lignin, and it is widely found in fruits, vegetable, whole grains and legumes [85]. Some epidemiological studies have shown that fiber intake in NAFLD patients is lower than in healthy individuals [86,87,88].

However, if we exclude the trials in which fiber was part of multifactorial dietary changes, only limited research regarding the effects of fiber alone on NAFLD has been done. We have found only one study evaluating the effects of a non-digestible carbohydrate, oligofructose (Table 3). In this trial, a decrease in ALT and AST was found after 16 g of oligofructose compared to maltodextrine in patients with NASH although no change in liver fat was detected at US [89].

Trying to draw some conclusions from the trials evaluating the effects of low GI diets on NAFLD, the few data available indicate that the low GI may have some role within the context of a diet characterized by other favorable changes such as, in primis, the reduction of saturated fatty acids.

#### 2.2.2. Fructose/Other Simple Sugars

The intake of simple sugars increases liver fat content in animal models [90,91] and epidemiological studies suggest an association between consumption of soft drinks and NAFLD development in humans [92,93,94].

Simple sugars, in particular fructose, has been shown to promote hepatic lipogenesis by stimulating SREBP-1c and carbohydrate response element-binding protein (ChREBP), the major transcription factors of many enzymes involved in de novo lipogenesis [95,96,97]. Furthermore, it has been observed that fructose and glucose consumption in addition to stimulating SREBP-1c and ChREBP may produce inflammation and activate cellular stress pathways [98,99] (Figure 2).

Many clinic trials have investigated the effect of simple sugars mainly fructose, glucose and sucrose on NAFLD in healthy individuals and in those at high cardiometabolic risk [100,101,102,103,104,105,106,107], and two meta-analyses on this issue were carried out [108,109]. Briefly, the first meta-analysis reported that, in healthy subjects, using high doses of fructose in terms of 104–220 g/day in a hypercaloric diet increased both liver fat content and serum ALT levels, while it did not produce any effect in isocaloric conditions [108]. Similar findings were reported by the second meta-analysis where it was observed that the excess of added sugar intake in a hypercaloric diet compared with a eucaloric control diet increased liver fat content [109].

Only three trials have looked specifically at the effect of simple sugars intake as part of an isocaloric diet in overweight/obese subjects (Table 3).

Johnston et al. investigated the effects of glucose- or fructose-sweetened beverages providing 25% of energy requirements during an isocaloric period of two weeks. At the end of treatment, in overweight patients with NAFLD, serum ALT and AST levels, and liver fat content evaluated by ^1^H-MRS were unchanged [110]. Similar findings were reported by Bravo et al. who investigated the effects of three different levels of sucrose or high-fructose corn syrup (55% fructose) at 8%, 18%, or 30% of the calories required for weight maintenance in overweight patients with NAFLD. At the end of a 10-week intervention, liver fat content evaluated by CT was unchanged [103]. On the other hand, Maersk et al. compared the effects of four different drinks 1 L/day of regular cola, or isocaloric semi-skim milk, or aspartame-sweetened diet cola or water in obese subjects with NAFLD. After 24 weeks of treatment, drinking regular cola resulted in a higher amount of liver fat content, evaluated by ^1^H-MRS. In particular, in pairwise comparisons, the increment was 143% compared with milk, 139% compared with diet cola, and 132% compared with water, despite the fact that total energy intake was not different between groups during the study, indicating that the consumption of regular cola and milk was compensated for by reducing the energy intake from other sources [102].

In conclusion, even if data are still limited, it seems that simple sugars, at least within the context of an isocaloric diet, do not have a marked deleterious influence on liver fat in overweight individuals, while frankly obese subjects may be more sensitive to the exposure to simple sugars even within an isocaloric diet.

### 2.3. Proteins

Limited evidence on the effect of proteins on NAFLD is available. In animal models, a reduction in liver fat content was observed when protein intake was increased [111].

A very recent analysis of The Rotterdam Study, a large epidemiological study, showed that total protein intake, in particular proteins of animal origin, was associated with higher odds of NAFLD in overweight subjects (OR = 1.50; 95% CI 1.17–1.92) [112]; similarly, a cross-sectional evaluation of the Israeli National Health and Nutrition Survey showed that the intake of meat was significantly associated with an increased risk for NAFLD (OR = 1.37, 95% CI 1.04–1.83) [113].

The effect of protein intake on NAFLD has been evaluated only in few controlled clinical trials, generally adopting hypocaloric diets [114,115,116]. Therefore, it is not possible to draw any conclusion about the possible effect of proteins per se on NAFLD.

### 2.4. Other Dietary Components

#### 2.4.1. Polyphenols

Polyphenols represent a great variety of secondary plant metabolites and, based on their chemical structure, they can be divided into two major categories: flavonoid and non-flavonoids. About 8000 phenolic compounds in the plant kingdom have been discovered. Vegetables, cereal grain, fruits, and some beverages—tea, coffee, red wine, and beer—are good sources of polyphenols [117]. Mean total dietary intake of polyphenols was 1193 ± 510 mg/day in a French cohort [118]. These natural compounds are powerful antioxidants, in addition to having many other properties such as anti-inflammatory, anti-mutagenic, and immunomodulatory activities [117].

Phenolic compounds have received growing interest over the last few years and epidemiological studies have shown an inverse correlation between high polyphenol consumption and incidence of many chronic metabolic diseases, including obesity, insulin resistance, and CVD [119]. A randomized controlled trial in individuals at high cardiometabolic risk showed that diets naturally rich in polyphenols improved fasting and postprandial dyslipidemia and reduced oxidative stress [120]. Recently, beneficial effects of polyphenols on NAFLD have been reported in animal models [121].

Polyphenols could prevent liver fat accumulation and NAFLD progression through several mechanisms. In both in vitro and animal models, it has been observed that polyphenols may reduce hepatic lipogenesis and increase free fatty acid oxidation. In particular, polyphenols can decrease the transcription of SREBP-1c [122] and increase transcription of PPAR-α. Moreover, polyphenols can improve insulin sensitivity and reduce the transcription of inflammatory cytokines [123,124,125]. All these molecular pathways can be indirectly modulated by the effect of polyphenols on the activation of AMP-activated protein kinase [126]. Finally, the antioxidant properties of phenolic compounds in reducing oxidative stress involved in NAFLD progression should be also considered [121] (Figure 2). Whereas from these animal and in vitro studies it can be argued that polyphenols may have positive influence on different aspects of NAFLD, the controlled intervention trials in humans have produced discordant results (Table 4).

The effects of mixed phenolic compounds have been investigated by two trials [127,128]. Chang et al. [127] evaluated in overweight NAFLD patients the effects of 150 mg/day of polyphenols composed of 1.43% flavonoids, 2.5% anthocyanins and 1.7% phenolic acids compared to placebo. After 12 weeks of treatment, in the polyphenol group a significant 15% reduction in fatty liver score was observed, with no changes in AST or ALT levels. In this group, a decrease in body weight, BMI, body fat and waist-to-hip ratio was also observed, possibly accounting, at least in part, for the reduction in fatty liver score. In a trial conducted by Guo et al. [128], young NAFLD patients evaluated by US were given 250 mL of bayberry juice or placebo twice daily for four weeks. The amount of polyphenols—anthocyanins and phenolic acids—was 1350 mg/day. No significant differences in the serum levels of AST and ALT between the groups were observed; however, a reduction in serum levels of hepatocytes apoptosis biomarkers, namely CK-18 and tissue polypeptide-specific antigen, was reported.

Two more trials evaluated the effects of specific polyphenols, anthocyanins and catechins, respectively [129,130]. In the first one, overweight NAFLD men were assigned to consume two bottles of purple sweet potato beverage or placebo. The amount of phenolic compounds represented by acylated anthocyanins was 400 mg/day. After eight weeks, the intake of purple sweet potato beverage induced a significant reduction in the serum levels of ALT versus placebo [129]. In the second study, Sakata et al. [130] investigated the effects of green tea with high-density catechins in overweight NAFLD patients evaluated with ultrasonography and computed tomography. Patients were randomized to consume different amounts of catechins, 0, 200, or 1080 mg/day, for 12 weeks in a cup of 700 mL/day. The consumption of the highest dose of catechins significantly decreased serum ALT level by 42.1% and improved liver fat content with a liver-to-spleen CT attenuation ratio that increased from 92% to 102%.

Resveratrol is currently one of the more studied polyphenols, and five trials have been conducted on NAFLD. In two of these studies, resveratrol at the dose of 500 mg/day and 1500 mg/day, respectively, did not induce any change in the different liver outcomes evaluated (liver fat content by imaging, liver markers, histological changes) [131,132]. In another intervention trial, where a higher dose of resveratrol was used 3000 mg/day a transient increase in ALT and AST was observed at Week 6, with no change at the end of the intervention in liver enzymes or in liver fat [133]; conversely, two trials showed some beneficial effects [123,134]. Chen et al. [123] observed a significant reduction in serum ALT and AST, fibroblast growth factor-21 and CK-18 after three months of treatment with 600 mg/day of resveratrol versus placebo, while no significant differences in hepatic fat content was found. Similarly, Faghihzadeh et al. [134] evaluated the effects of 500 mg/day of resveratrol versus placebo in overweight patients for 12 weeks. After the intervention period, serum levels of ALT and AST were significantly decreased in both groups, although in patients on resveratrol a significantly greater reduction in ALT (−32.3%, *p* = 0.03) was found. Furthermore, in the resveratrol group a significant reduction in CK-18 was observed as well as an improvement in the hepatic tissue echogenicity. Nevertheless, the transient elastography did not show a reduction in hepatic fibrosis grade.

Therefore, based on these data, catechins and antocianins seem to have some beneficial influence on liver fat, but this needs to be corroborated by additional evidence. As for resveratrol, the results are so discordant that no definite conclusion can be drawn.

#### 2.4.2. Caffeine

Caffeine, an alkaloid xanthine derivate, represents the main compound of coffee, the most consumed beverage worldwide, and can also be found in some food, tea and soft drinks.

Over the last few years, epidemiological studies have shown an inverse correlation between high coffee or caffeine consumption and incidence of many chronic metabolic diseases, even if many studies have been unable to evidence if the beneficial effect is related to coffee or caffeine [135]. In several prospective and cross sectional studies, the relationship between coffee/caffeine consumption and NAFLD has been investigated and we report the comprehensive results of three meta-analyses published in the last year [136,137,138]. It should be considered that controlled clinical trials have not been performed so far.

Wijarnpreecha et al. reported a 29% significant reduction in the risk of developing NAFLD in patients who drank coffee (RR, 0.71; 95% CI, 0.60–0.85) and a 30% decreased risk of developing liver fibrosis [136]. Marventano et al. reported an inverse association of caffeine intake with fibrosis levels in four of seven studies on NAFLD fibrosis [137], while Shen et al. reported that only regular coffee/caffeine intake, i.e., ingestion of caffeine only from regular coffee, not including other caffeinated beverages such as soda, tea, espresso, etc., was significantly associated with reduced hepatic fibrosis in two out of three studies also analyzed by Marventano [138]. In conclusion, most of the evidence on coffee/caffeine and NAFLD suggest that coffee more than caffeine may have beneficial effect on fibrosis, however, it should be considered some limitations of these studies: the definition of regular coffee consumption widely varied between studies; furthermore, these are meta-analyses of observational studies that could only show an association, but not a causal relationship. With respect to the plausible mechanisms, it has been reported that caffeine may reduce the progression of liver fibrosis by inhibiting hepatic stellate cell adhesion and activation [139]. Furthermore, it is important to underline that other coffee compounds present in coffee such as polyphenols and melanoidins could positively influence NAFLD [140].

#### 2.4.3. Vitamin E

The term vitamin E refers to eight lipid-soluble compounds four tocotrienols and four tocophenols with powerful antioxidant properties. These essential vitamins are synthesized in vegetables and are largely present in seeds, nuts, vegetable oils, green leafy vegetables and fortified cereals [141]. In terms of vitamin E pharmacokinetics it is important to underline that all the eight isoforms reach the liver, but only the α-tocopherol form for the selective binding offered by the α-tocopherol transfer protein is retained at high levels in hepatocytes, whereas other vitamin E forms are preferentially metabolized by microsomal P450 or excreted into the bile [142].

Vitamin E plays a key role in many physiological functions: it is one of the most powerful antioxidant and acts as free radical scavenger; it is also involved in the regulation of platelet aggregation, protein kinase C activation, immune function, gene expression, and other metabolic processes [143].

Oxidative stress and inflammation are the major contributors to NAFLD progression, and vitamin E could play an important role in mitigating oxidative stress. Vitamin E could avoid the progression of NAFLD and improve NASH by virtue of its antioxidant capacity and as free radical scavenger. It has been observed that vitamin E reduces the inflammatory pathway in NASH by several mechanisms, beyond its “simple” antioxidant activity; in particular, vitamin E could improve superoxide dismutase activity and could decrease the transcription of many genes related to inflammation and liver fibrosis [144,145,146,147]; it has been also reported that vitamin E could improve insulin sensitivity [148] (Figure 2).

The possible effects of vitamin E supplementation on NAFLD have been assessed in different intervention trials and the results of these trials have been examined in two meta-analyses [149,150]. Briefly, vitamin E supplementation in patients with NAFLD reduces significantly liver enzymes, liver steatosis, inflammation and hepatocellular ballooning compared to control treatments. Moreover, in patients with NASH, vitamin E supplementation seems to reduce fibrosis as well. Despite the positive results of these meta-analyses, it is important to underline some limitations such as the variability in daily dosage of vitamin E, the length of treatment, and the small sample size of the studies, except for the PIVENS and the TONIC studies [151,152]. Furthermore, some concerns must be underlined about the possible negative effects of high doses of vitamin E (>400 IU/day) on all-cause mortality [153].

#### 2.4.4. Vitamin C

Vitamin C is a soluble vitamin and its major dietary forms l-ascorbic and dehydroascorbic acids are largely found in vegetables and fresh fruits [154]. Vitamin C plays a key role in many physiological functions for human health; it is essential for the activity of the enzymes implicated in the synthesis of catecholamines, carnitine and collagen; and it is a powerful antioxidant and acts as a free radical scavenger [155].

In the last few years, noteworthy epidemiological literature has shown an inverse correlation between vitamin C deficiency and some chronic diseases, as obesity, hypertension, and CVD [156]. The results of epidemiological studies are conflicting about a possible relation between vitamin C and NAFLD. In fact, Ferolla et al. [157] reported that patients with NAFLD were unable to achieve the optimal intake of vitamin C, and similar findings were reported by Musso et al. [47] and Canbakan et al. [158], who analyzed the intake of vitamin C in patients with NASH. Conversely, in other cross-sectional studies no relation between dietary vitamin C intake and presence of NAFDL or NASH was observed [159,160,161]. These conflicting results may be related to ethnicity and differences in disease grade (NAFL or NASH); furthermore, it should be considered that in many studies the dietary intake of vitamin C was considered with no evaluation of plasma vitamin C levels.

Theoretically, vitamin C could play a beneficial role in NAFLD by acting as powerful antioxidant and as free radical scavenger. In in vitro models, vitamin C can reduce reactive oxygen species formation and improve the activity of glutathione peroxidase and superoxide dismutase [162]. Furthermore, vitamin C can promote the production of adiponectin an adipose tissue protein apparently able to decrease insulin resistance and inflammation in humans [163] (Figure 2).

To the best of our knowledge, no clinical trial has investigated the effect of vitamin C supplementation alone on NAFLD, while some clinical trials have evaluated the effects of the combination of vitamins C and E (Table 5). The results of these trials were not concordant, two studies showing a reduction in fibrosis and NAFLD activity score evaluated on liver biopsy [164,165], two studies showing no effects on liver fat [166,167].

#### 2.4.5. Vitamin D

Vitamin D is a lipid-soluble compound found in few foods, such as fatty fish, fish liver oils, and dairy products; it is also produced in the skin after ultraviolet irradiation. Vitamin D_2_ and vitamin D_3_, also called ergocalciferol and cholecalciferol, are the two main forms of vitamin D. Vitamin D plays a prominent role in calcium and phosphorus metabolism and is essential for bone health, promoting bone growth and remodeling. In the last decade, it has become evident that vitamin D also presents extra-skeletal effects, including metabolic effects, neuromuscular and immune functions [168].

A growing body of literature has shown that serum levels of vitamin D are inversely associated with insulin resistance, metabolic syndrome, CVD, diabetes and NAFLD [169,170,171]. A meta-analysis showed that subjects with NAFLD were 26% more likely to present vitamin D deficit than controls [172]. Vitamin D receptors are widely expressed in the liver and can explain the possible effect of vitamin D on NAFLD. Vitamin D may down-regulate the expression of the NF-κB involved in the transcription of inflammatory cytokines and improve the expression of PPAR-α in the liver [173]. Furthermore, it has been observed that vitamin D increases adiponectin secretion and decreases lipolysis in adipose tissue [174], improves the expression of GLUT-4 receptor in skeletal muscle [175] and promotes insulin secretion [176]. All these effects mediated by the specific vitamin D receptor could reduce liver fat content (Figure 2).

To date, very few clinical trials have investigated the effects of vitamin D supplementation on NAFLD (Table 6).

Two of them [177,178] showed no effect of vitamin D supplementation on liver enzymes, liver fat content or hepatic biomarkers of injury and fibrogenesis, i.e., CK-18 and *N*-terminal Procollagen III Propeptide. In the third one [179], the effect of vitamin D supplemented to a hypocaloric diet was evaluated compared to a hypocaloric diet. Liver enzymes and liver fat content evaluated by US were significantly reduced by vitamin D independently of weight loss, which was similar in the two groups.

## 3. Conclusions

NAFLD is the most common chronic liver disease in the industrialized world and will become one of the most important public health challenges in the coming decades for its hepatic and extra-hepatic related complications. At present, lifestyle intervention including hypocaloric diet and regular physical exercise represents the mainstay of NAFLD management. Apart from the caloric restriction alone, changes in the quality of the diet modulating either the macro- or the micronutrient composition can also markedly affect the clinical evolution of NAFLD offering a more realistic and feasible alternative for NAFLD treatment. We tried to review the relevant data available from intervention studies in humans. Unfortunately, data are rather scant and characterized by methodological limitations, and the results are often discordant.

Notwithstanding, the following conclusions can be drawn:

1. Data are reasonably convincing for the possible effects of dietary macronutrients on liver fat content. In fact, SFA increase liver fat content and replacing SFA with MUFA or *n*-6 PUFA reduces liver fat, while the effectiveness of *n*-3 PUFA supplementation is still controversial.
2. In terms of other dietary components (polyphenols) and micronutrients, data are not yet convincing, and any effect would refer especially to liver inflammation and fibrosis more than to fat content. Only for vitamin E supplementation, data are more convincing, even if concerns may be present for high vitamin E supplementation considering possible negative effects on all cause-mortality.

Similar conclusions can also be drawn for NAFLD in children and adolescents [180].

Therefore, based on the available evidence in humans, precise recommendations on the quality of diet to be used for the prevention and treatment of NAFLD may not be given. More carefully conducted intervention studies are needed: it is very likely that the “optimal diet” for NAFLD should be based on different dietary modifications, i.e., a multifactorial diet, able to act both on the deposition of excess fat in the liver and on the other pathways leading from liver fat deposition to NASH and fibrosis. However, this hypothesis needs to be substantiated by appropriate intervention studies in humans.

## Figures and Tables

**Table 1:** Clinical trials on the effects of MUFA and *n*-6 PUFA on NAFLD in individuals at high cardiometabolic risk.

Author (Reference)	Study Design	Study Population Participants Age BMI	Intervention and Doses	Duration Weeks	Observed Effects with MUFA or *n*-6 PUFA			
					Liver Imaging	Liver Biomarkers	Liver Scores	Liver Biopsy
**MUFA**								
Bozzetto et al., 2012 [53]	Randomized, controlled, parallel group	36 M/F, T2DM	MUFA diet (MUFA 28% TE) vs. high-CHO/fiber/low GI diet ( MUFA 16% TE)	8	↓ LIVER FAT (^1^H-MRS)	AST =	n.a.	n.a.
		58.7 years				ALT =		
		29.7 kg/m^2^						
Ryan et al., 2013 [54]	Randomized, controlled, crossover	12 M/F, T2DM	Mediterranean diet (MUFA 23% TE) vs. low fat-high CHO (MUFA 8% TE )	6	↓ LIVER FAT (US)	AST =	n.a.	n.a.
		55.0 years				ALT =		
		32.0 kg/m^2^						
Nigam et al., 2014 [55]	Randomized, controlled, parallel group	93 M	olive oil (MUFA 70%) vs. canola oil (MUFA 61%) vs. soybean or safflower oil (MUFA 15–24% TE)	24	↓ LIVER FAT (US)	AST =	n.a.	n.a.
		37.0 years				ALT =		
		27.4 kg/m^2^						
***n*-6 PUFA**								
Bjermo et al., 2012 [64]	Randomized, controlled, parallel group	61 M/F	PUFA diet (linoleic acid 15% TE) vs. SFA diet (butter 15% TE)	10	↓ LIVER FAT (^1^H-MRS)	AST n.a.	n.a.	n.a.
		56.5 years				ALT =		
		30.2 kg/m^2^						

TE: total energy; = no changes; ↓ significant decrease. BMI: body mass index; MUFA: monounsaturated fatty acids; *n*-6 PUFA: *n*-6 polyunsaturated fatty acids; vs.: versus; T2DM: type 2 diabetes mellitus; M: male; F: female; CHO: carbohydrates; GI: glycemic index; SFA: saturated fatty acids; ^1^H-MRS: proton magnetic resonance spectroscopy; ALT: alanine aminotransferase; AST: aspartate aminotransferase; n.a.: not assessed; US: ultrasonography.

**Table 2:** Clinical trials on the effects of *n*-3 PUFA on NAFLD in individuals at high cardiometabolic risk.

Author (Reference)	Study Design	Study Population Participants Age BMI	Intervention and Doses	Duration Weeks	Observed Effects with *n*-3 PUFA			
					Liver Imaging	Liver Biomarkers	Liver Scores	Liver Biopsy
Spadaro et al., 2008 [66]	Parallel group randomized, controlled	36 M/F	2 g/day vs. placebo	24	↓ LIVER FAT (US)	AST =	n.a.	n.a.
		50.1 years				ALT ↓		
		30.5 kg/m^2^						
Scorletti et al., 2014 [67]	Double-blind, placebo-controlled	103 M/F	4 g/day (EPA 1.8 g, DHA 1.5 g) vs. placebo	72	↓ LIVER FAT (MRI)	AST =	n.a.	n.a.
		51.5 years				ALT =		
		33.0 kg/m^2^						
Zhu et al., 2008 [68]	Double-blind, placebo-controlled	134 M/F	6 g/day vs. placebo	24	↓ LIVER FAT (US)	AST =	n.a.	n.a.
		44.5 years				ALT ↓		
		26.2 kg/m^2^						
Vega et al., 2008 [69]	Crossover placebo-controlled	16 M/F	9 g/day (EPA 51.4%, DHA 23.9%) vs. placebo	8	= LIVER FAT (^1^H-MRS)	AST n.a.	n.a.	n.a.
		50.0 years				ALT n.a.		
		36.2 kg/m^2^						
Argo et al., 2015 [70]	Double-blind, placebo-controlled	34 M/F	3 g/day (EPA 35%, DHA 25%) vs. placebo	48	= LIVER FAT (MRI)	AST =	n.a.	= NASH score
		46.8 years				ALT =		
		32.5 kg/m^2^						
Sanyal et al., 2014 [71]	Double-blind, placebo-controlled	243 M/F	EPA 1.8 g/day vs. EPA 2.7 g/day vs. placebo	48	n.a.	AST =	n.a.	= NASH score
		48.7 years				ALT =		
		34.8 kg/m^2^						
Cussons et al., 2009 [72]	Crossover placebo-controlled	25 F	4 g/day (EPA 27%, DHA 56%) vs. placebo (oleic acid 67%)	8	= LIVER FAT (^1^H-MRS)	AST n.a.	n.a.	n.a.
		54.5 years				ALT =		
		34.8 kg/m^2^						

= no changes; ↓ significant decrease. BMI: body mass index; M: male; F: female; *n*-3 PUFA: *n*-3 polyunsaturated fatty acids; US: ultrasonography; n.a.: not assessed; MRI: magnetic resonance imaging; ALT: alanine aminotransferase; AST: aspartate aminotransferase; DHA: docosahexaenoic acid; EPA: eicosapentaenoic acid; ^1^H-MRS: proton magnetic resonance spectroscopy; NASH: non-alcoholic steatohepatitis.

**Table 3:** Clinical trials on the effects of different types of carbohydrates (low glycemic index diet, oligofructose, and simple sugars) on NAFLD in individuals at high cardiometabolic risk.

Author (Reference)	Study Design	Study Population Participants Age BMI	Intervention and Doses	Duration Weeks	Observed Effects with Carbohydrates, Oligofructose and Simple Sugars			
					Liver Imaging	Liver Biomarkers	Liver Scores	Liver Biopsy
**Low Glycemic Index Diet**								
Fraser et al., 2008 [84]	Open label, quasi-randomized, controlled	259 M/F	ADA diet (CHO: 50–55%, fat: 30%, protein: 20%) or LGI diet (CHO: 50–55%, fat: 30%, protein: 15–20%) or MM diet (CHO: 35%, fat: 45%, protein: 15–20%)	52	n.a.	ALT ↓	n.a.	n.a.
		T2DM						
		56 years						
		31.5 kg/m^2^						
Utzschneider et al., 2012 [81]	Randomized parallel, double-bind	35 M/F	LSAT diet (23% fat, 7% saturated fat, GI < 55) vs. HSAT diet (43% fat, 24% saturated fat, GI > 70)	4	↓ LIVER FAT (^1^H-MRS)	AST =	n.a.	n.a.
		68.9 years				ALT =		
		27.5 kg/m^2^						
Ramon-Krauel et al., 2013 [83]	Randomized parallel	16 M/F	LGI diet (CHO: 40%, fat: 35–40%, protein: 15–20%) vs. LF diet (CHO: 55–60%, fat: 30%, protein: 15–20%)	24	= LIVER FAT (^1^H-MRS)	AST =	n.a.	n.a.
		12.8 years				ALT =		
		32.6 kg/m^2^						
Misciagna et al., 2016 [82]	Randomized parallel-group, controlled	98 M/F	LGI diet (CHO: 50%, fat: 30%, protein: 15–20%) vs. control (diet based on INRAN guidelines)	24	↓ LIVER FAT (US)	AST =	n.a.	n.a.
		47.5 years				ALT =		
		31.5 kg/m^2^						
**Oligofructose**								
Daubiol et al., 2005 [89]	Randomized double-blind, crossover controlled	7 M	Oligofructose (16 g/day) vs. maltodextrine	8	= LIVER FAT (US)	AST ↓	n.a.	n.a.
		NASH				ALT =		
		54.5 years						
		29.1 kg/m^2^						
**Fructose/Simple Sugars**								
Johnston et al., 2011 [110]	Randomized double-blind	32 M	Fructose (25% TE) vs. Glucose (25% TE)	2	= LIVER FAT (^1^H-MRS)	AST =	n.a.	n.a.
		33.9 years				ALT =		
		29.4 kg/m^2^						
Bravo et al., 2013 [103]	Randomized parallel-group	64 M/F	HFCS (8%, 18% or 30% of the calories required for weight maintenance) vs. Sucrose (8%, 18% or 30% of the calories required for weight maintenance)	10	= LIVER FAT (CT)	n.a.	n.a.	n.a.
		42.1 years						
		27.2 kg/m^2^						
Maersk et al., 2012 [102]	Randomized parallel-group	47 M/F	Regular cola (1 L/day) or Milk (1 L/day) or Diet cola (1 L/day) or Water (1 L/day)	24	↑ LIVER FAT (^1^H-MRS)	n.a.	n.a.	n.a.
		38.7 years						
		32.0 kg/m^2^						

TE: total energy; = no changes; ↓ significant decrease; ↑ significant increase. BMI: body mass index; M: male; F: female; T2DM: type 2 diabetes mellitus; ALT: alanine aminotransferase; AST: aspartate aminotransferase; n.a.: not assessed; ADA: American Diabetes Association; CHO: carbohydrates; LGI: low glycemic index; MM: Mediterranean modified; US: ultrasonography; ^1^H-MRS: proton magnetic resonance spectroscopy; CT: computed tomography; LGI: low glycemic index; INRAN: Italian National Research Institute for Foods and Nutrition; LSAT: low-fat/low-saturated fat/low-glycemic index diet; HSAT: high-fat/high-saturated fat/high-glycemic index diet; GI: glycemic index; LF: low fat; HFCS: high-fructose corn syrup.

**Table 4:** Clinical trials on the effects of polyphenols supplementation on NAFLD in individuals at high cardiometabolic risk.

Author [Reference]	Study Design	Study Population Participants Age BMI	Intervention and Doses	Duration Weeks	Observed Effects with Polyphenols			
					Liver Imaging	Liver Biomarkers	Liver Scores	Liver Biopsy
Suda et al., 2008 [129]	Double-blind, randomized, placebo-controlled	38 M	Anthocianins (400 mg/day) vs. placebo	8	n.a.	AST n.a.	n.a.	n.a.
		43.0 years				ALT ↓		
		25.4 kg/m^2^						
Sakata et al., 2013 [130]	Double-blind, randomized, placebo-controlled	17 M/F	Cathechin (1.080 mg/day) vs. placebo	12	↓ LIVER FAT (CT)	AST n.a.	n.a.	n.a.
		50.6 years				ALT ↓		
		29.0 kg/m^2^						
Chang et al., 2013 [127]	Double-blind, randomized, placebo-controlled	36 M/F	Flavonoids, anthocyanins, phenolic acid (150 mg/day) vs. placebo	12	↓ LIVER FAT (US)	AST =	↓ FS	n.a.
		37.9 years						
		31.2 kg/m^2^				ALT =		
Guo et al., 2014 [128]	Double-blind, randomized, crossover, placebo-controlled	44 M/F	Phenolic acids, anthocyanins (1350 mg/day) vs. placebo	4	n.a.	AST =	n.a.	n.a.
		21.2 years						
		25.4 kg/m^2^				ALT =		
						TPS ↓		
						CK-18 ↓		
Poulsen et al., 2013 [131]	Double-blind, randomized, placebo-controlled	24 M	Resveratrol (500 mg/day) vs. placebo	4	= LIVER FAT (^1^H-MRS)	AST n.a.	n.a.	n.a.
		38.3 years				ALT =		
		34.2 kg/m^2^						
Faghihzadeh et al., 2014 [134]	Double-blind, randomized, placebo-controlled	50 M/F	Resveratrol (500 mg/day) vs. placebo	12	↓ LIVER FAT (US) = LIVER FIBROSIS (TEL)	AST = ALT ↓	n.a.	n.a.
		45.1 years				CK-18 ↓		
		28.5 kg/m^2^						
Chychay et al., 2014 [133]	Double-blind, randomized, placebo-controlled	20 M	Resveratrol (3000 mg/day) vs. placebo	8	= LIVER FAT (^1^H-MRS)	AST * ↑	n.a.	n.a.
		48.1 years				ALT * ↑		
		31.5 kg/m^2^				CK-18 =		
Chen et al., 2014 [123]	Double-blind, randomized, placebo-controlled	60 M/F	Resveratrol (600 mg/day) vs. placebo	12	= LIVER FAT (US)	AST ↓	n.a.	n.a.
		44.3 years						
		25.7 kg/m^2^				ALT ↓		
						CK-18 ↓		
						FGF-21↓		
Heebøll et al., 2016 [132]	Double-blind, randomized, placebo-controlled	28 M	Resveratrol (1500 mg/day) vs. placebo	24	= LIVER FAT (^1^H-MRS)	AST =	n.a.	= NASH
		(46% NASH)						
		43.3 years				ALT =		
		31.9 kg/m^2^						

* at Week 6; = no changes; ↓ significant decrease; ↑ significant increase. BMI: body mass index; M: male; F: female; ALT: alanine aminotransferase; AST: aspartate aminotransferase; n.a.: not assessed; US: ultrasonography; CT: computed tomography; ^1^H-MRS: proton magnetic resonance spectroscopy; TEL: transient elastography; FS: fatty liver score; TPS: tissue polypeptide-specific antigen; CK-18: Cytokeratin-18; FGF-21: fibroblast growth factor 21; NASH: non-alcoholic steatohepatitis.

**Table 5:** Clinical trials on the supplementation of vitamins C + E on NAFLD in individuals at high cardiometabolic risk.

Author (Reference)	Study Design	Study Population Participants Age BMI	Intervention and Doses	Duration Weeks	Observed Effects with Vitamin C Plus Vitamin E			
					Liver Imaging	Liver Biomarkers	Liver Scores	Liver Biopsy
Harrison et al., 2003 [164]	Double-blind, randomized, placebo-controlled	45 M/F, NASH	Vitamin C (1000 mg/day) + Vitamin E (1000 IU/day) vs. placebo	24	n.a.	AST =	n.a.	=NASH ↓ FIBROSIS
		51.3 years				ALT =		
		32.7 kg/m^2^						
Ersöz et al., 2005 [166]	Open-label, randomized	57 M/F	Vitamin C (500 mg/day) + Vitamin E (600 IU/day) vs. UDCA (10 mg/kg/day)	24	= LIVER FAT (US)	AST =	n.a.	n.a.
		(15% NASH)						
		47.1 years				ALT =		
		28.4 kg/m^2^						
Nobili et al., 2006 [167]	Double-blind, randomized, placebo-controlled	90 M/F	Vitamin C (500 mg/day) + Vitamin E (600 IU/day) vs. placebo	52	= LIVER FAT (US)	AST =	n.a.	n.a.
		(26% NASH)						
		12.1 years				ALT =		
		25.0 kg/m^2^						
Nobili et al., 2008 [165]	Open-label, randomized, placebo-controlled	53 M/F	Vitamin C (500 mg/day) + Vitamin E (600 IU/day) vs. placebo	104	n.a.	AST =	n.a.	=LIVER FAT = NASH = FIBROSIS ↓ NAFLD activity score
		11.9 years						
		25.8 kg/m^2^				ALT =		

= no changes; ↓ significant decrease. BMI: body mass index; NASH: non-alcoholic steatohepatitis; M: male; F: female; ALT: alanine aminotransferase; AST: aspartate aminotransferase; n.a.: not assessed; UDAC: ursodeoxycholic acid; IU: international unit; US: ultrasonography; NAFLD non-alcoholic fatty liver disease.

**Table 6:** Clinical trials on the effects of vitamin D supplementation on NAFLD in individuals at high cardiometabolic risk.

Author (Reference)	Study Design	Study Population Participants Age BMI	Intervention and Doses	Duration Weeks	Observed Effects with Vitamin D			
					Liver Imaging	Liver Biomarkers	Liver Scores	Liver Biopsy
Sharifi et al., 2014 [177]	Double-blind, randomized, placebo-controlled, parallel	53 M/F	Cholecalciferol (3570 IU/day) vs. placebo	16	= LIVER FAT (US)	AST =	n.a.	n.a.
		42.1 years				ALT =		
		30.3 kg/m^2^						
Barchetta et al., 2016 [178]	Double-blind, randomized, placebo-controlled	65 M/F	Cholecalciferol (2000 IU/day) vs. placebo	24	= LIVER FAT (^1^H-MRS)	AST =	= FLI	n.a.
		T2DM						
		58.6 years				ALT =		
						CK-18 =		
		30.0 kg/m^2^				P3NP =		
Lorvand Amiri et al., 2016 [179]	Double-blind, randomized, placebo-controlled	73 M/F	Cholecalciferol (1000 IU/day) + hypocaloric diet vs. placebo + hypocaloric diet	12	↓ LIVER FAT (US)	AST ↓	n.a.	n.a.
		41.9 years				ALT ↓		
		30.3 kg/m^2^						

= no changes; ↓ significant decrease; BMI: body mass index; M: male; F: female; T2DM: type 2 diabetes mellitus; ALT: alanine aminotransferase; AST: aspartate aminotransferase; n.a.: not assessed; US: ultrasonography; ^1^H-MRS: proton magnetic resonance spectroscopy; IU: international unit; CK-18: Cytokeratin-18; P3NP: N-terminal Procollagen III Propeptide; FLI: fatty liver index.

## Abbreviations

The following abbreviations are used in this manuscript:

AHA	American Heart Association
ALA	α-linolenic acid
ALT	alanine aminotransferase
AST	aspartate aminotransferase
BMI	body mass index
ChREBP	carbohydrate response element-binding protein
CK-18	cytokeratin-18
CT	computed tomography
CVD	cardiovascular disease
DHA	docosahexaenoic acid
EPA	eicosapentaenoic acid
GL	glycemic load
GI	glycemic index
^1^H-MRS	Proton magnetic resonance spectroscopy
MUFA	monounsaturated fatty acids
NAFL	non-alcoholic fatty liver
NAFLD	non-alcoholic fatty liver disease
NASH	non-alcoholic steatohepatitis
PNPLA3	patatin-like phospholipase 3
PPAR-α	peroxisome proliferator-activated receptor-alfa
PPAR-γ	peroxisome proliferator-activated receptor-gamma
PUFA	polyunsaturated fatty acids
SFA	saturated fatty acids
SREBP-1c	sterol regulatory element-binding transcription factor-1c
TD2M	type 2 diabetes mellitus
TM6SF2	transmembrane 6 superfamily member 2
US	ultrasonography
